# Measles outbreak linked to European B3 outbreaks, Wales, United Kingdom, 2017

**DOI:** 10.2807/1560-7917.ES.2017.22.42.17-00673

**Published:** 2017-10-19

**Authors:** Jonny Currie, Llion Davies, Joanne McCarthy, Malorie Perry, Catherine Moore, Simon Cottrell, Mererid Bowley, Chris Williams, Ananda Giri Shankar, Rhianwen Stiff

**Affiliations:** 1 Public Health Wales, Cardiff, Wales, United Kingdom; 2 Aneurin Bevan Gwent Public Health Team, Abertillery, Wales, United Kingdom

**Keywords:** United Kingdom, Wales, air-borne infections, viral infections, measles, outbreaks

## Abstract

The United Kingdom achieved interrupted endemic measles transmission for 36 months in 2016. Despite this, ongoing challenges from sporadic measles cases typically imported from abroad remain. We summarise a B3 measles genotype outbreak in south-east Wales occurring between May and September 2017, linked with other European outbreaks, and lessons learnt. Seventeen confirmed cases and one probable case occurred principally in education and healthcare-settings. Six confirmed cases attended healthcare settings when infectious, without being isolated.

In May 2017, Wales’ national health protection service began receiving measles notifications from the Newport and Torfaen areas in south-east Wales. Seventeen cases were confirmed over 4 months from education and healthcare settings. We present here epidemiological and microbiological findings from outbreak investigations and summarise lessons for health protection teams facing similar outbreaks.

## Case confirmation and laboratory testing

Following the first notifications, members of the health protection team began categorising cases according to agreed case definitions, designed specifically for this outbreak (Box). We gathered contact information from healthcare professionals or via direct interview and recorded data on an EpiData database [1]. Recorded details included case status, demographics, vaccination status, exposure information, travel history and information regarding symptoms. We produced case-contact network diagrams in Cytoscape [2]. Vaccination coverage for the affected areas was calculated using data from the National Community Child Health Database in Wales.

Box **Definition of cases and other categories followed-up during the measles outbreak, Wales, United Kingdom, May–September 2017**

**Cases **

***Possible case***

i. any person with fever, generalised maculopapular rash and one of cough/coryzal symptoms/conjunctivitis;

ii: any person with an epidemiological link to a laboratory-confirmed case of measles with unverified acute illness including a rash.

***Probable case***

Any person meeting the clinical criteria under (i) **AND** with an epidemiological link to a laboratory-confirmed case.

***Confirmed case***

Any person meeting the clinical criteria and with laboratory confirmation of measles.

**Other categories**

***Population at risk***

Those resident in Newport and Torfaen, two neighbouring local authority areas in south-east Wales.

***Contact***

Any person living in the population at risk areas who was exposed to a probable or confirmed case during the infectious period.

***Epidemiologically linked***

Contacts who became cases if exposure occurred between 7 and 21 days before rash onset.

From each notified case we used a dual sampling and testing protocol. For investigation of cases, we undertook both a dry buccal swab, sent to the local laboratory for a rapid measles RT-PCR testing and an oral fluid sample, sent to the World Health Organization (WHO) measles reference laboratory in London for confirmation and genotyping as previously described [3].

## Epidemiology and microbiological findings

Initial cases were notified in May 2017 in Newport, with subsequent cases in Torfaen. Newport and Torfaen are adjacent local authority areas in south-east Wales comprising urban and semi-rural geographies and with populations 149,148 and 92,052 respectively [4]. The outbreak comprised 17 confirmed cases. One probable and three possible cases (Table) did not submit samples for testing. Data presented here relate to all cases including those meeting confirmed, probable and possible definitions.

**Table:** Description of cases, measles outbreak linked to European B3 outbreaks, Wales, United Kingdom, 2017 (n = 21)

Characteristic	Classification	n
Case status	Confirmed	17
	Probable	1
	Possible	3
Sex	Male	7
	Female	14
School age^a^	Yes	14
	No	7
Vaccination status	0 MMR	13
	1 MMR	2
	2 MMR	6
Admitted to hospital	Yes	5
	No	16
At-risk group^b^	Yes	5
	No	12
Contacts^c^ per case (range)	Mean	4.2 (2–7)

MMR: measles-mumps-rubella.

^a^ Aged 4 to 18 years.

^b^ Immunosuppressed, pregnant women, neonate or healthcare worker.

^c^ Confirmed contacts only. Contacts are defined as confirmed (known relationship to a case) or unconfirmed. Unconfirmed contacts are those which are either: (i) recorded on the Welsh Demographic Service database as living at the same address as a case but the household members have not confirmed that they are living there or (ii) the individual was in the same place as a case at the same time but it is not known whether they meet the contact definition.

**Figure 1:**
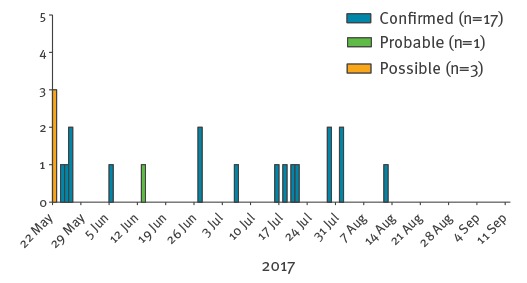
Onset dates for cases in the outbreak, by case status^a^, measles outbreak linked to European B3 outbreaks, Wales, United Kingdom, 2017 (n = 21)

^a^ All three cases marked as possible have a proxy date of onset which is the approximate date first off school with a rash

**Figure 2:**
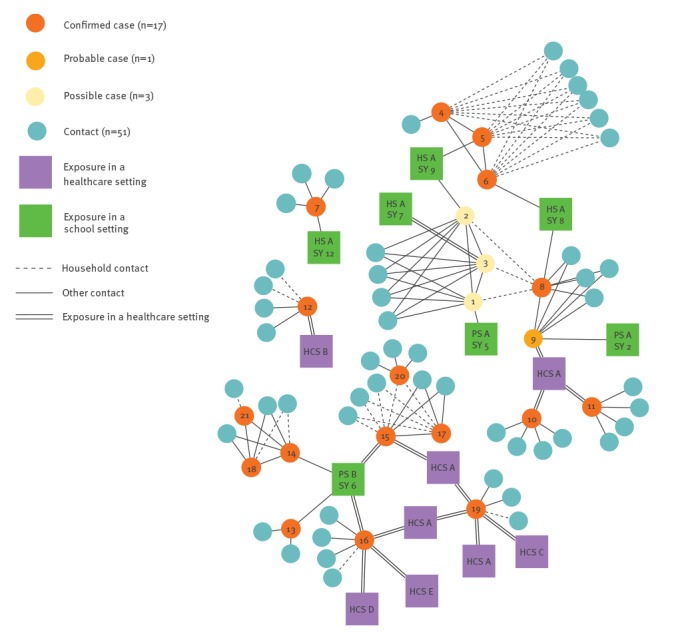
Contact network for cases, measles outbreak linked to European B3 outbreaks, Wales, United Kingdom, 2017 (n = 21)

The outbreak proceeded steadily, with new cases emerging in line with expected incubation periods of 7-21 days (Figure 1). Epidemiological investigations revealed a likely source from whom onward transmission to our index case occurred, however this individual was not available for testing. The mean age of cases was 11.5 years (range: 11 months–29 years). Transmission settings included a high school in four contiguous school years, two primary schools and a hospital emergency department (Figure 2). The health protection team discovered that six of the confirmed cases had attended healthcare settings during their infectious period without being isolated. The healthcare settings included a paediatric department, an obstetric clinic, an oncology centre, a dental surgery and a GP surgery. Transmission of measles during these exposures in healthcare settings resulted in three new confirmed cases (2 children below the vaccination age and 1 healthcare worker). Investigation following emergence of healthcare-associated measles cases revealed generally incomplete and sparse occupational health records of staff vaccination histories.

HCS A: hospital A, paediatrics; HCS B: hospital B; HCS C: hospital C; HCS D: dental practice; HCS E: general practice; HS: high school; PS: primary school; SY: school year.

The numbers in the circles represent the case numbers as per line list.

This diagram summarises interactions between cases and their contacts revealed during the epidemiological investigations of the outbreak control team. The key on the left describes the categorisation of cases and the setting in which exposure occurred, including household, school and healthcare facility. Names of institutions have been removed to protect individual confidentiality.

Genotyping results confirmed that the outbreak was caused by a genotype B3 measles virus. Molecular epidemiology demonstrated evidence of onward community transmission and confirmed that the virus was genetically identical to the B3 virus circulating in mainland Europe [5,6].

## Control measures

The management of the outbreak was overseen by a multi-agency outbreak control team (OCT) comprising Public Health Wales, local authorities/education, local health boards and primary care. The health protection team rapidly investigated notifications and instigated active case finding, providing exclusion advice and organising testing. Contacts were risk-assessed with vulnerable individuals offered post-exposure prophylaxis as per national guidelines [7]. Where cases had attended educational or healthcare settings during their likely infectious period, communications were issued and those with significant exposure were risk assessed and managed appropriately. Existing relationships between health and education agencies allowed rapid dissemination of multi-lingual information to at risk communities via a variety of methods: postal, short message service (SMS), school Internet pages. Wider communications were also circulated to local healthcare providers to facilitate case finding and infection control measures.

Local vaccination data on resident children estimated Newport and Torfaen measles-mumps-rubella (MMR)1 coverage to be 93.5% and 94.7% respectively, and full MMR coverage to be 85.9% and 92.3%, respectively. However, coverage varied according to age and area of residence. Additionally, it became apparent that there were children in the schools who were completely unknown to the health system. They were recent migrants to the area from abroad and had not been registered with general practitioners (GPs), a factor known to be linked with poor vaccination uptake [8]. There was no information on their vaccination status available to either the school nursing team or the child health systems. Aneurin Bevan University Health Board, supported by the Local Education Authority and local public health team, organised MMR vaccination sessions in 60 primary and secondary schools over 16 weeks, with 1,238 vaccinations administered to date. Primary care was also asked to identify all under vaccinated children, between the ages of 1 year and 19 years, and invite them for vaccination. Support was also offered by the Health Board to primary care to achieve this.

Over the course of this outbreak, the OCT met on 19 occasions, issued 12 press releases and posted 57 social media messages in Welsh and in English with 5,457 user interactions across Facebook and Twitter during the outbreak period. Media messages encouraged where appropriate vaccination and advised individuals with symptoms suggestive of measles to contact healthcare settings before attending. Media messages were also shared across Wales to help prevent onward transmission beyond the outbreak area.

## Discussion

By the end of 2016, 33 of the 53 WHO European region countries had demonstrated elimination of endemic transmission of measles over a period of 36 consecutive months; the United Kingdom in particular passed this threshold in 2016 [9]. The legacy in Wales of the discredited and retracted 1998 Lancet article by Wakefield et al. [10] was a precipitous reduction in MMR vaccination to ca 70% at the age of 5 years [11]; while vaccination uptake has since improved (89.7% in children age 5 years since 2013) coverage of two MMR doses falls short of the 95% target required for herd immunity and pockets of under-vaccination remain, leaving individuals and communities at risk [12], and unlike some countries [13], MMR vaccination is not mandatory for school enrolment in Wales.

This outbreak of 17 confirmed and one probable measles cases in Wales was epidemiologically linked with ongoing outbreaks across Europe and originated in a community with low uptake of MMR vaccination with particular issues regarding registration with primary care. Given the eternal challenge of maintaining population resilience to measles following elimination of endemic transmission, presenting the factors assisting in controlling this outbreak provides a number of lessons that may be of value to other health agencies.

Firstly, provision of catch-up vaccination sessions in schools during outbreaks is feasible and effective, as large numbers of previously unvaccinated pupils were vaccinated. Though studies have alluded to challenges in vaccinating school-aged children [14-16], our approach of close collaboration with schools, historically strong relationships between local public health teams and local education authority colleagues and of merging school pupil and primary care lists to identify children in need of vaccination led to greater numbers being protected against measles. Developing these relationships before an outbreak and maintaining them over time seems to us key for any health agency aiming to avert a future large-scale measles outbreak.

Secondly, our initial cases were from a cluster of unvaccinated children attending the same school, highlighting a gap in existing surveillance and vaccination systems. Systems that identify under-vaccinated children on school enrolment could be made more effective. Electronic data linkage between primary care, child health and education agencies with routine updates would facilitate this approach, an area we are exploring and hope to share lessons on in the future.

Thirdly, this outbreak built on our prior experience of social media in health protection. Our media communications demonstrated significant reach across those platforms we used, as shown by user engagement. Evaluation of these communications revealed even larger figures for the number of times our content was viewed. However, one limitation of such media to our knowledge is the inability to assess impact in practice: whether individuals acted upon advice disseminated via media is unclear. Thus, while we believe it important to utilise such digital tools in outbreaks, the importance of traditional opportunities to engage with individuals and communities should not be overlooked.

Finally, following investigation of exposures in healthcare settings it was noted that healthcare staff vaccination records were occasionally unavailable or incomplete. Measles outbreaks have been reported in healthcare settings in several European countries [5,17], demonstrating the need for healthcare staff to be adequately immunised and aware of protocols for swift isolation of suspected cases. Though Department of Health guidance has long proclaimed it essential for frontline healthcare staff to be protected with MMR vaccine [18], in practice it appears this guidance is not being implemented, nor is MMR as yet mandatory for staff. Given the risk of transmission in healthcare settings identified in this outbreak, all front line healthcare staff should be reminded that they should check their vaccination and immunity status. Furthermore, they should be kept informed and reminded of the need to isolate potential measles cases upon arrival.
